# The Role of Na/K-ATPase Signaling in Oxidative Stress Related to Obesity and Cardiovascular Disease

**DOI:** 10.3390/molecules21091172

**Published:** 2016-09-03

**Authors:** Krithika Srikanthan, Joseph I. Shapiro, Komal Sodhi

**Affiliations:** 1 Department of Medicine, Joan C. Edwards School of Medicine, Marshall University, Huntington, WV 25701, USA; srikanthan.krithika@gmail.com (K.S.); shapiroj@marshall.edu (J.I.S.); 2 Department of Surgery, Joan C. Edwards School of Medicine, Marshall University, Huntington, WV 25701, USA

**Keywords:** Na/K-ATPase, oxidative stress, pNaKtide, obesity, cardiovascular disease

## Abstract

Na/K-ATPase has been extensively studied for its ion pumping function, but, in the past several decades, has been identified as a scaffolding and signaling protein. Initially it was found that cardiotonic steroids (CTS) mediate signal transduction through the Na/K-ATPase and result in the generation of reactive oxygen species (ROS), which are also capable of initiating the signal cascade. However, in recent years, this Na/K-ATPase/ROS amplification loop has demonstrated significance in oxidative stress related disease states, including obesity, atherosclerosis, heart failure, uremic cardiomyopathy, and hypertension. The discovery of this novel oxidative stress signaling pathway, holds significant therapeutic potential for the aforementioned conditions and others that are rooted in ROS.

## 1. Introduction

**Figure 1:**
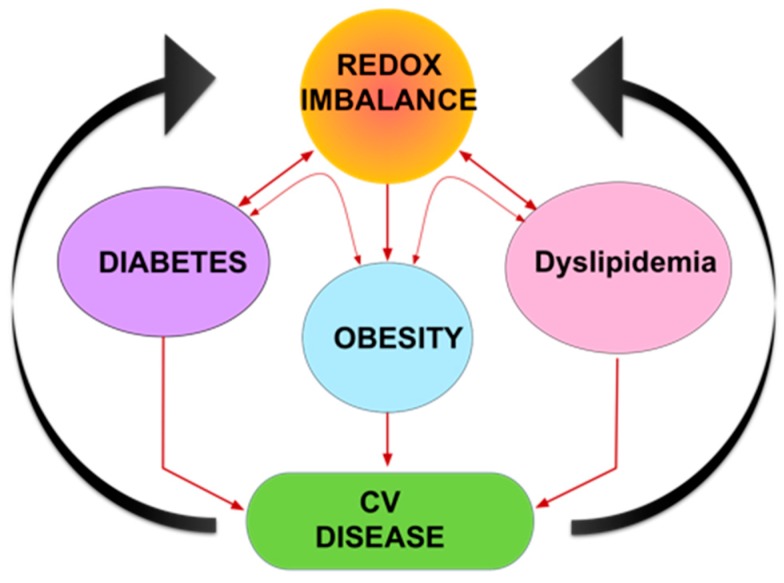
Oxidative Stress Imbalance. Redox imbalance is central to the pathophysiology of chronic disorders, including obesity, metabolic syndrome, and cardiovascular diseases, such as atherosclerosis, and diabetes. These disorders are intertwined in a vicious, feed-forward loop of oxidative stress, which eventually leads to the development of end organ damage that is frequently seen with chronic disorders like these.

The Na/K-ATPase enzyme (“sodium pump”) is well known for its ion pumping function, but was also discovered to have a scaffolding and signaling function by Dr. Zijian Xie in the late 1990s. Over the last twenty years, studies have shown that this signaling pathway, and alterations with it, is involved with a number of clinical disorders, including cancer, chronic kidney disease, and cardiovascular diseases, such as uremic cardiomyopathy [1,2]. Our recent work with obesity models has provided evidence that the Na/K-ATPase signaling cascade activation worsens obesity, diabetes, dyslipidemia, and atherosclerosis, as these conditions are all related to an imbalance of oxidative stress (Figure 1) [3]. Na/K-ATPase and the signaling pathway present increasing importance, given the therapeutic potential it holds for the aforementioned clinical disorders.

## 2. Na/K-ATPase: Structure, Function, and the Xie Model of Signaling

Na/K-ATPase primarily consists of three subunits denoted by α, β, and γ, with α and β subunits necessary for ion pumping. The α subunit contains the ATP, digitalis, and other ligand binding sites, and is considered the “catalytic subunit” [1]. The Na/K-ATPase α subunit, with 10 transmembrane domains, has four isoforms. The α1 isoform is found in all cells, whereas the α2 and α3 isoforms are expressed in skeletal muscle, neuronal tissue, and cardiac myocytes. Na/K-ATPase belongs to the type-II class of P-type ATPases, and contains four distinct functional domains. The A domain consists of the N-terminus and the first cytoplasmic loop connects to transmembrane helices M2 and M3. Although there is great sequence variation at the N-terminus between SERCA (sarco/endoplasmic reticulum Ca^2+^-ATPase) and the Na/K-ATPase, both enzymes appear to have the same two α-helix motifs. Most importantly, based on the structure of SERCA, the A domain is highly exposed for binding of other proteins. The enzyme also has the highly conserved phosphorylation (P) domain that is close to the membrane and a relatively isolated nucleotide-binding (N) domain. The structure–function relationship of Na/K-ATPase was extensively studied in the latter portions of the 20th century, and has received new attention due to the recently recognized Na/K-ATPase scaffolding and signaling functions.

### Xie Model of Na/K-ATPase Signaling

The Xie model for the Na/K-ATPase signaling function was derived from difficulties explaining signaling with the ionic model, along with experimental observations regarding reactive oxygen species (ROS) and tyrosine kinase activities being critical to such signaling. This model proposed that the caveolar Na/K-ATPase α1 subunit serves as a negative regulator of Src and that during conformational changes in α1 induced by CTS or oxidation, Src is allowed to become active and trigger a signal cascade, which involves the generation of ROS. The α1 subunit of the Na/K-ATPase binds Src and appears to maintain it in an inactive state. However, binding CTS appears to alter the Na/K-ATPase structure allowing Src to become activated, which, in turn, transactivates the EGFR, and begins the signal cascade, which causes increases in ROS.

**Figure 2:**
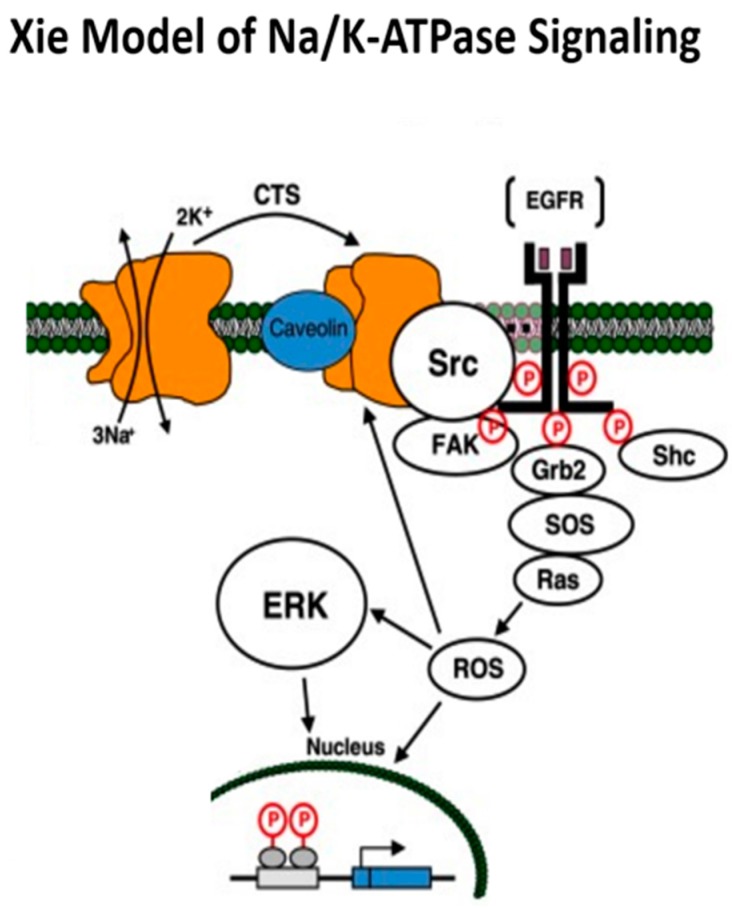
Xie Model of Na/K-ATPase Signaling. In this model, cardiotonic steroids (CTS) induce the Na/K-ATPase signal cascade, which eventually leads to the development of ROS. We suggest that in the microdomain of caveolae, the Na/K-ATPase functions as a scaffolding protein to interact with CTS and change conformation to activate Src. Src then transactivates the EGFR, which leads to a signal cascade involving FAK, Shc, Grb2 and SOS resulting in the generation of ROS, which in turn activates additional Na/K-ATPase molecules, as well as causes downstream activation of ERK and effects nuclear transcription. Abbreviations: Epidermal growth factor (EGFR); focal adhesion kinase (FAK); Src homology 2 domain protein (Shc); growth factor receptor bound protein 2 Grb2); son of sevenless protein (SOS); extracellular-signal-regulated kinase (ERK).

The Na/K-ATPase-Src complex appears to function similar to a receptor tyrosine kinase. It appears that there is a critical binding of the tyrosine kinase domain of Src by a portion of the N domain of the α1 subunit. Under basal conditions, this binding inhibits the tyrosine kinase function of Src. We speculate that conformational changes induced in Na/K-ATPase by cardiotonic steroids and/or the specific oxidation of some amino-acids (vida infra) result in the internalization of this epitope and the disinhibition of the tyrosine kinase function of Src with attendant downstream signaling. Downstream activation of PLC, PI3K and PKC has also been established. This model is shown schematically in Figure 2, and in our admittedly biased opinion, constitutes an important advance in our understanding of sodium pump signaling [1].

In recent years, several studies have challenged Xie’s model of direct interaction of c-Src and the α1 subunit as this interaction is critical to CTS induced signaling. In Xie’s model, the α1 subunit provides the ligand binding site and the associated c-Src functions as the kinase moiety. The following evidence supports the notion that Na/K-ATPase directly interacts with Src to form a functional receptor complex in live cells [4,5,6,7,8]. Several labs have observed that Na/K-ATPase and Src were co-enriched in caveolar fractions in different types of cells. Immunofluorescence has shown co-localiztion of c-Src and the α1 subunit in the plasma membrane. FRET (fluorescence resonance energy transfer) and BRET (bioluminescence resonance energy transfer) analysis indicated that c-Src and the α1 were directly interacting in close proximity. Co-immunoprecipitation assays demonstrated direct contact of c-Src and the α1 subunit. This subsequently received scrutiny because detergents could denature Na/K-ATPase and interactions could be just the interaction of the unfolded Na/K-ATPase subunits and Src [9]. However, Tian et al. actually performed the immunoprecipitation assays both with and without detergents to control for exactly this [4]. GST pulldown assays demonstrated direct interactions between the α1 subunit and Src. They further went on to dissect which domains of Src interact with Na/K-ATPase using in vitro binding assays, and found that the Src SH2 domain binds to α1 CD2 domain and the Src kinase domain associates with the α1 N domain [10].

Those who challenged Xie’s model, propose that c-Src activation is primarily due to an ATP-sparing effect based on cell-free sytem [9,11,12]. Based on Src activity assays performed on pig kidney cell free mixtures, Gable et al. concluded there that was “no solid evidence for direct molecular interation of Src with Na/K-ATPase under physiological conditions” [9]. However, similar to Xie [4], they found that Src-418 phosphorylation was reduced when both Na/K-ATPase and Src were present in a reaction mixture and that the addition of ouabain partially antagonized the inhibitory effect of Na/K-ATPase on Src phosphorylation. Unlike Xie, Gable et al. found that vanadate increased Src phosphorylation (as much as ouabain), indicating that such activation is due to the ATP-sparing effects of ATPase inhibitors. Yosef et al. performed pulldown assays between recombinant purified human Src kinase and α1β1FXYD1 and published findings which do not support the direct binding of Src to Na/K-ATPase in 2016, however they did corroborate a direct caveolin 1 and Na/K-ATPase interaction in renal membranes [13]. Another study with contradictory results to Xie was by Clifford et al. who were unable to show a oubain induced interaction between c-Src and α1 by immunoprecipiration assays in human breast tumor and non-tumorigenic cells [14].

At this time, we are not able to fully explain the dissimilarities in results between all research groups, but there are noticeable differences in experimental design(different cell lines and animal models used) at the very least. The Src family consists of at least nine members and many of them are expressed in a tissue specific manner. The interaction differences we are seeing may constitute the tissue specific receptor Na/K-ATPase/Src family kinase complex that provides a certain degree of signaling specificity [15]. Another reason for the discrepancies is that there is still much that is unknown about the Na/K-ATPase/Src signaling complex, which is why further studies are warranted to resolve this controversial topic.

## 3. Oxidative Stress and Regulation of the Na/K-ATPase

Binding of ouabain to Na/K-ATPase activates Src kinase, which in turn transactivates EGFR, and leads to the activation of the Ras-Raf-MEK-ERK pathway. Ras activation leads to the activation of MAPK and an increase in [Ca^2+^], which results in the opening of mitochondrial ATP-sensitive K^+^ channels and generation of mitochondrial ROS. Additionally, through the Na/K-ATPase/c-Src signaling cascade, c-Src activation regulates NOX (NADPH oxidase)-derived superoxide generation [16]. Conversely, studies have shown that oxidative stress can also activate Na/K-ATPase signaling in addition to being produced by this pathway.

Increases in ROS can oxidize the Na/K-ATPase α/β subunits and its independent regulator FXYD proteins. This oxidation inhibits its activity and promotes its susceptibility to degradation by proteasomal and endosomal/lysosomal proteolytic pathways [17,18,19,20,21,22,23]. It appears that the oxidized modification of Na/K-ATPase is usually a reversible modification [19], however, ROS can accelerate degradation of the oxidatively damaged Na/K-ATPase [20].

Mechanisms of redox-induced regulation of Na/K-ATPase activity include S-glutathionylation, S-nitrosylation, carbonylation, phosphorylation, and 4-hydroxynonenal exposure; oxygen-derived free radicals, H2O2, NO, and oxidized glutathione are the signaling messengers that make Na/K-ATPase “redox sensitive” in different cell types.

S-glutathionylation of the β1 subunit has been shown to cause a modest, reversible, decrease in Na/K-ATPase activity in rabbit cardiomyocytes, Xenopus oocytes, and pig kidneys [24,25]. Exposure to oxidized glutathione results in the presence of S-glutathionylated cysteine residues in the α1 subunit. This results in a dose and time dependent suppression of Na/K-ATPase function in duck salt glands, rabbit kidneys, and rat myocardium. [26]. In both α1 and β1 S-glutathionylation, inhibition of Na/K-ATPase activity occurs via stabilizing the enzyme in the E2 prone conformation or by blocking the ATP-binding site.

S-nitrosylation is the modification of the cysteine thiol by incorporation of a NO moiety. S-nitrosylation of Cys46 in the β-subunit of Na/K-ATPase was reported to cause a 20% decrease in Na/K-ATPase activity upon exposing cardiomyocytes to hypoxia. Furthermore, S-nitrosylation has been shown to be necessary as an intermediate step in the induction of regulatory S-glutathionylation of the β-subunit of Na/K-ATPase [24,25].

Protein carbonylation is another process, which results in the alteration of Na/K-ATPase function in conditions of oxidative stress [27]. Yan et al. demonstrated that both, CTS and glucose oxidase-induced H2O2, stimulates direct α1 subunit carbonylation of Pro222 of the actuator(A) domain and this is involved in a reversible, feed-forward mechanism of regulation of renal proximal tubule Na/K-ATPase signal transduction [28]. Similarly, in vivo studies have demonstrated that in obesity models (increased systemic oxidative stress), there is also increased α1 subunit carbonylation in visceral adipose tissue, which is accompanied by increased Na/K-ATPase pathway signal transduction-increased phosphorylated c-Src and ERK expression [3].

FXYD proteins associate with the Na/K-ATPase αβ complex, stabilizing it and modulating the enzyme activity. These modulatory subunits, also known as phospholemman (PLM), may undergo phosphorylation, however, it is unphosphorylated PLM which has been shown to decrease Na/K-ATPase function [29]. On the other hand, PKC phosphorylation of Ser36 and Ser68 detaches PLM inhibition from Na/K-ATPase and increases Na/K-ATPase signaling activity. PLM also contain cysteine residues that may undergo reversible S-glutathionylation, similar to the α and β subunits. Thus, interaction between the subunits and the enzyme function is affected by S-glutathionylation of the PLM [30].

Finally, 4-hydroxynonenal (4-HNE), a lipid peroxidation product, is another oxidative stress byproduct, which has been shown to inhibit Na/K-ATPase activity, via reacting with sulfhydryl groups irreversibly [31,32]. Interestingly, Yamaguchi et al. demonstrated decreased α1 protein expression, but no change with β1 expression, with exposure to 4-HNE in spiral ligament fibrocytes [33]. In summary, Na/K-ATPase activity and regulation is extremely sensitive to changes in the redox state. Additionally, while the breadth of literature of how this enzyme is regulated via oxidative mechanisms continues to grow, it needs to be investigated in greater depth.

## 4. The Development of pNaKtide

The development of pNaKtide is detailed in our JBC 2009 publication [34]. We identified a portion of the α1 subunit that could interface with the Na/K-ATPase kinase domain and determined an effective peptide (NaKtide), which we then merged with a TAT leader sequence to facilitate cellular permeability. In addition to making the peptide cell permeable, it also appears to localize the peptide to the membrane component of the cell, restricting its inhibition to the membrane associated portion of Src. Our data suggest that this results in pNaKtide altering ouabain induced signaling events while not affecting signal cascades, which are distinct from membrane associated Src. However, this does not mean that pNaKtide is specific for ouabain or other CTS stimulated pathways, since we have data suggesting it is effective in disease states where CTS are not implicated (discussed below). pNaKtide, however, was not the only Src inhibitor. Pyrrolopyrimidines have been reported as potent inhibitors of c-Src with inhibitory potency at concentrations as low as 10 nM. Other c-Src kinase inhibitors include quinolinones, which have potencies for in vitro c-Src inhibition at 500 nM, iminochromenes with a potency of 2 μM, and isothiazolones, which are effective at 4 μM for inhibition of the c-Src family kinase, p56lck [35]. Herbimycin A and CP-118,556 are also two reagents, which have been shown to exhibit specificity for inhibition of c-Src. Herbimycin A, a benzoquinone ansamycin antibiotic, inhibits Src family kinases by disrupting Src interactions with heat shock proteins (especially HSP90). CP-118,556, a pyrazolopyrimidine, interacts specifically with Src family kinases and is a competitive inhibitor of ATP, like most other tyrosine kinase inhibitors, which are ATP mimetics [36].

PP2 (4-amino-5-[4-chlorophenyl]-7-[*t*-butyl] pyrazolo [3,4-d]pyrimidine) is a well-known generic Src inhibitor, which has been comparatively studied with pNaKtide. Unlike PP2, pNaKtide resides in the membranes, and therefore, pNaKtide is effective in disrupting the formation of the Na/K-ATPase/Src receptor complex in a dose-dependent manner. pNaKtide consequently blocks ouabain-induced activation of Src, ERK, and hypertrophic growth in cardiac myocytes. Prior studies by Li et al. have compared pNaKtide and PP2 and while both pNaKtide and PP2 have a similar IC50 on Src kinase, PP2 produces more inhibition on basal Src activity than that of pNaKtide in both LLC-PK1 and cardiac myocytes [34]. However, the key difference between PP2 and pNaKtide is that pNaKtide appears to more specifically target the pool of Na/K-ATPase-interacting Src and has fewer (off-target) effects than PP2. This was particularly salient to our group since our ultimate goal is to study the Na/K-ATPase/Src signaling pathway, specifically, as it leads to ROS production and ultimately oxidative stress related conditions, such as obesity, NASH, atherosclerosis, and diabetes. Other Src inhibitors do not have the specificity to the Na/K-ATPase complex that pNaKtide does, therefore they do not afford us the opportunity to study this novel pathway in depth [37].

## 5. Role of Oxidative Stress and Na/K-ATPase/ROS Signaling in Obesity

**Figure 3:**
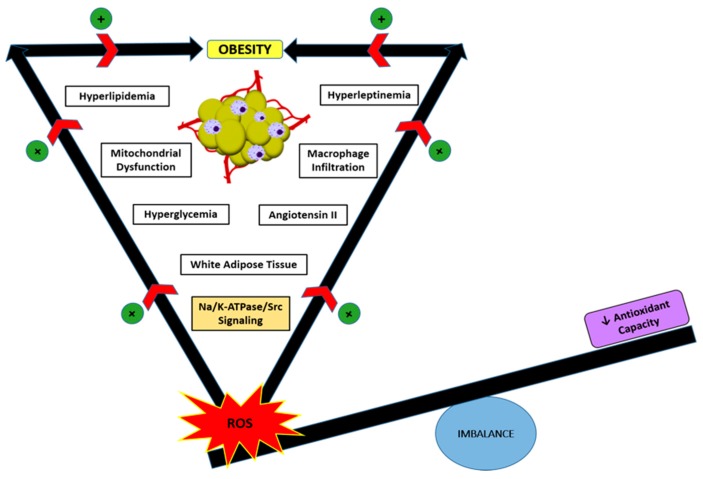
Systemic imbalance of prooxidants vs. antioxidants in obesity. Sources of ROS in the obese state, including the Na/K-ATPase/Src signaling pathway, engage in a positive feedback loop to produce more ROS, in a setting of limited antioxidant capacity, thereby worsening the existing obesity and comorbid conditions.

Obesity is now a worldwide epidemic, with approximately 400 million considered obese (BMI ≥ 30) (World Health Organization, Geneva 2006), and poses a major risk for metabolic syndrome and cardiovascular related mortality [38]. The imbalance of prooxidants and antioxidant defenses (Figure 3) results in systemic oxidative stress and this is known to play a role in the generation and maintenance of the obesity phenotype in both isolated adipocytes and intact animals [39,40,41,42]. Studies have shown that fat accumulation (obesity) is correlated with systemic oxidative stress in both humans and mice [43]. Well-known oxidative injury markers, such as MDA (malondialdehyde), lipid hydroperoxides, conjugated dienes, 4-HNE (4-hydroxynonenal), 8-epi-PGF2α, and TBARS (thiobarbituric acid reactive substances), have been positively correlated with obesity in human clinical studies [44,45]. Studies in mouse models of obesity have shown that the production of ROS increases selectively in white adipose tissue (WAT) of obese mice, suggesting that in obesity, increased oxidative stress in plasma is due to increased ROS production from accumulated fat [43,46,47]. The WAT, and not other tissues, of obese mice also has augmented expression of NADPH oxidase (one of the major sources of ROS in adipocytes) and decreased expression of antioxidant enzymes [43]. In cultured adipocytes, ROS production is markedly increased during differentiation of 3T3-L1 cells into adipocytes, suggesting that ROS production increases in parallel with fat accumulation in adipocytes [43,46]. This is supported by the fact that increasing levels of free fatty acids [48] can induce ROS production through NAPDH oxidase activation. Further, ROS can upregulate mRNA expression of NADPH oxidase, establishing a viscous cycle of more ROS production [43].

Adipocyte mitochondrial dysfunction is also proposed as one of the reasons there is increased ROS production in obesity [38,46]. In mouse models of obesity, the mitochondrial population is approximately 50% lower in white adipocytes from obese mice, compared to control mice. Furthermore, about 50% of gene transcripts encoding mitochondrial proteins are decreased with the onset of obesity in white adipocytes from obese mice compared to control mice [49].

ROS derived from hypertrophied adipocytes may also initiate an inflammatory state in WAT establishing a feedback loop between inflammation and oxidative stress in obese adipose tissue furthering WAT dysfunction [46]. Macrophages infiltrate the obese adipose tissues and are an important source of inflammatory cytokines and possibly augment NADPH oxidase activity and ROS production. In vitro studies have established that adipocytes exposed to ROS upregulate expression of proinflammatory cytokines and macrophage chemoattractive molecules, both of which are able to stimulate more ROS production [50,51], once again highlighting another cycle of ROS production.

There are also several metabolic parameters and complications, which are dysregulated in obesity, and contribute to and amplify oxidative stress. Hyperglycemia induces oxidative stress through activation of the polyol and hexosamine pathways, production of advanced glycation end-products (AGE), and increase of diacylglycerol (DAG) synthesis. Hyperlipidemia induces ROS formation by increasing lipid oxidation and protein carbonylation. Leptin and angiotensin II, both of which are secreted by adipocytes, are inducers of ROS generation. Leptin promotes inflammation and lipid peroxidation, while angiotensin II stimulates AT1R-NADPH oxidases. Dysregulation of all of these metabolic parameters with adipose tissue expansion contributes to oxidative stress [45,46].

Finally, an obese state is also associated with reduced antioxidant defenses in adipocytes. For examples, WAT from obese mouse models have decreased expression and activity of antioxidant enzymes such as SOD, GPX, and catalase [43]. Obese people have low levels of total antioxidants characterized by lower levels of serum vitamins A, E, C and β-carotene, as well as glutathione [46]. Collectively, the subpar ROS scavenging system might exacerbate the high levels of ROS produced by hypertrophied adipocytes.

Similarly, our work has focused on targeting a novel oxidative stress signaling pathway which may balance the prooxidant and antioxidant scales back to equilibrium—the Na/K-ATPase/Src/ROS amplification loop. Because Na/K-ATPase can amplify oxidant signaling, we speculated that the novel peptide, pNaKtide, might ameliorate the obesity phenotype. To test this hypothesis, we performed studies in 3T3-L1 preadipocytes and found that pNaKtide attenuated oxidant stress and lipid accumulation in a dose-dependent manner. Complementary experiments in C57Bl6 mice fed a high-fat diet corroborated our in vitro observations. We demonstrated the ability of the Na/K-ATPase signal cascade to amplify ROS involved in adipogenesis, a process not previously linked to cardiotonic steroids or the Na/K-ATPase signal cascade. Administration of pNaKtide in these mice reduced body weight, restored systemic redox and inflammatory milieu, and, crucially, improved insulin sensitivity. Inhibition of the Na/K-ATPase amplification of oxidative stress may ultimately be a novel way to combat obesity, insulin resistance, and metabolic syndrome. With this background, we propose that visceral adipocytes create systemic oxidant stress through the feed-forward oxidant amplification loop of Na/K-ATPase-Src-EGFR signaling [3].

## 6. Role of Oxidative Stress and Na/K-ATPase/ROS signaling in Cardiovascular Disease (CVD)

CVDs are a class of pathologies that affect the heart primarily but also include vascular diseases of the brain, kidney and peripheral arterial system [52]. CVD is the leading cause of mortality in Western countries, with approximately 30% of deaths in the United States attributed to it and according to the World Health Organization; CVD is the principle cause of death globally [48,52,53]. In the vascular system, under physiological conditions, ROS are produced in low concentrations and act as a signaling molecule that regulate endothelial function and vascular tone by controlling vascular smooth muscle cell (VSMC) contraction and relaxation and participate in VSMC growth [54]. Under pathophysiological conditions, ROS play important roles in various cardiovascular complications including atherosclerosis, hypertension, congestive heart failure, ischemic heart disease, and ischemia-reperfusion injury. There are several potential sources of ROS production in CVD including NADPH oxidases (Nox family of enzymes), xanthine oxidase, mitochondrial dysfunction, and uncoupled nitric oxide synthases [54] and we suggest, the Na/K-ATPase-Src-EGFR signaling pathway as well.

Oxidative stress plays a central role in the pathogenesis of atherosclerosis, but is also known to occur in a state of chronic inflammation [55]. Oxidative stress affects transcription factors (such as NF-xB), which is inactivated by antioxidants and anti-inflammatory agents, suggesting that atherosclerosis is an inflammatory process, which is strongly affected by oxidative stress [56,57,58]. ROS produced by endothelial, vascular smooth muscle, and adventitial cells are detrimental to vascular function by direct injury to cell membranes and nuclei (direct endothelial dysfunction), interacting with endogenous vasoactive mediators formed in endothelial cells, modulating vasomotion, and via lipid peroxidation leading to the formation and accumulation of oxidized LDL, one of the key mediators of atherosclerosis. Our recent in vivo studies have demonstrated that the Na/K-ATPase/Src/ROS amplification loop is activated by diet-induced oxidative stress, contributing to the development and progression of dyslipidemia and atherosclerosis. We have shown that the novel pNaKtide has been able to mitigate both dyslipidemia and atherosclerosis through attenuation of c-Src and ERK1/2 signaling, which is corroborated with improved plasma ROS and TBARS levels. However, more interestingly, Na/K-ATPase pathway activation and increased oxidant stress happen despite there being no change in body weight in an ApoE knockout mouse model of atherosclerosis. This further demonstrates the importance of the Na/K-ATPase pathway in oxidant stress in CVD and the utility of equilibrating oxidant stress.

There is both direct and indirect evidence of increased oxidative stress in humans with heart failure. Patients with heart failure have elevated levels of plasma MDA and pericardial 8-isoprostane. Similar to obesity, mitochondrial dysfunction, xanthine oxidase, and NAPDH oxidase (Nox4 gene upregulation) activity is increased, are sources of ROS, and are associated with heart failure [54,59,60,61]. With regard to Na/K-ATPase, endomyocardial biopsies have demonstrated that patients with compromised heart function have total Na/K-ATPase concentrations decreased by 40%, and at the subunit level, the α1-, α3-, and β1-proteins levels are reduced in human heart failure [62]. Left ventricular hypertrophy (LVH) and diastolic dysfunction are frequent comorbidities in patients with chronic renal failure. SERCA2a is a sarcoplasmic reticulum calcium ATPase responsible for the rapid reduction of cytosolic calcium following systole and is downregulated in experimental chronic renal failure associated LVH [63,64]. Oxidant stress has also been implicated in the pathogenesis of uremic CVD [65]. Our group has extensive evidence that blockade of Na/K-ATPase signaling results in decreased oxidant stress, downregulation of SERCA2a and cardiac fibrosis in experimental uremic cardiomyopathy models [66,67,68,69].

Almost all experimental models of hypertension display some form of oxidative stress. While, all vascular cell types produce ROS, NADPH oxidase and mitochondrial enzymes seem to be particularly important in the development of ROS related to hypertension [70]. Given that renal function is a key determinant of blood pressure, the ability of oxidative stress to induce hypertension stems in part from effects on the renal vasculature and nephron [71]. Activation of the renin-angiotensin system is a major mediator of NADPH oxidase activation and ROS production in human hypertension [72]. Renal oxidative stress is associated with glomerular damage, proteinuria, sodium and volume retention, and nephron loss, all of which are important in the development of hypertension [73,74]. Coordinated regulation of two major ion transporters, the basolateral Na/K-ATPase and the apical NHE3, has been implicated in high salt intake (volume expansion) mediated blood pressure increase. Interestingly, the impairment of the Na/K-ATPase/c-Src signaling cascade has been shown to contribute to experimental Dahl salt-sensitive hypertension. The effect and interaction of ROS and Na/K-ATPase on renal proximal tubule sodium reabsorption has been explored in a limited capacity [71], so, moving forward, it will be key to explore whether ROS signaling is a link between the Na/K-ATPase/c-Src cascade and NHE3 regulation [16].

## 7. Conclusions

The Na/K-ATPase/Src signaling pathway highlighted here is a novel mechanism by which ROS may be produced in pathological conditions related to oxidative stress, such as obesity and cardiovascular disease. We have much to learn about the mechanisms by which the Na/K-ATPase/Src signaling pathway contributes to oxidative stress in obesity and CVD, but it is clear, thus far, that ROS produce, maintain and enhance these diseases states in a positive feedback loop, exacerbating the redox imbalance. Our novel peptide, pNaKtide, has significant promise in its potential to break the positive feedback loop of ROS amplification and may have broader applications for other oxidative stress related pathologies.
